# Frequency of Human Endogenous Retroviral Sequences (HERV) K113 and K115 in the Polish Population, and Their Effect on HIV Infection

**DOI:** 10.1371/journal.pone.0077820

**Published:** 2013-10-28

**Authors:** Katarzyna Zwolińska, Brygida Knysz, Jacek Gąsiorowski, Monika Pazgan-Simon, Andrzej Gładysz, Maciej Sobczyński, Egbert Piasecki

**Affiliations:** 1 Laboratory of Virology, Ludwik Hirszfeld Institute of Immunology and Experimental Therapy, Polish Academy of Sciences, Wrocław, Poland; 2 Department and Clinic of Infectious Diseases, Liver Diseases and Acquired Immune Deficiencies, Wrocław Medical University, Poland; 3 Department of Genomics, Faculty of Biotechnology, University of Wrocław, Poland; 4 Institute of Chemistry, Environmental Protection and Biotechnology, Jan Długosz University, Częstochowa, Poland; University of California San Francisco, United States of America

## Abstract

**Background:**

The human genome contains about 8% of endogenous retroviral sequences originated from germ cell infections by exogenous retroviruses during evolution. Most of those sequences are inactive because of accumulation of mutations but some of them are still capable to be transcribed and translated. The latter are insertionally polymorphic HERV-K113 and HERV-K115. It has been suggested that their presence and expression was connected with several human diseases. It is also believed that they could interfere with the replication cycle of exogenous retroviruses, including HIV.

**Results:**

Prevalence of endogenous retroviral sequences HERV-K113 and HERV-K115 was determined in the Polish population. The frequencies were found as 11.8% for HERV-K113 and 7.92% for HERV-K115. To verify the hypothesis that the presence of these HERVs sequences could affect susceptibility to HIV infection, comparison of a control group (HIV-negative, not exposed to HIV; n = 303) with HIV-positive patients (n = 470) and exposed but uninfected (EU) individuals (n = 121) was performed. Prevalence of HERV-K113 and HERV-K115 in the EU group was 8.26% and 5.71%, respectively. In the HIV(+) group we detected HERV-K113 sequences in 12.98% of the individuals and HERV-K115 sequences in 7.23% of the individuals. There were no statistically significant differences between groups studied.

**Conclusion:**

The frequency of HERV-K113 and HERV-K115 sequences in Poland were found to be higher than usually shown for European populations. No relation between presence of the HERVs and HIV infection was detected.

## Introduction

Human endogenous retroviruses (HERV) are probably remnants of ancient retroviral infections in human germ cell lines. According to different works they comprise about 8% of the human genome [1], [2], [3]. It is estimated that such insertions occurred 30–45 million years ago in the case of the “old” sequence HERV-W and HERV-K, and 150 thousand years ago in the case of “young” sequences such as HERV-K106 [4]–[6]. HERV structure is typical for retroviruses; they consist of *gag*, *pol* and *env* genes surrounded by long terminal repeat (LTR) sequences [7], [8]. *Gag* encodes the virion structural proteins (group specific antigens), *pol* encodes reverse transcriptase and protease, ribonuclease H (RNase H), and integrase, and the *env* gene encodes envelope proteins [9], [10]. The majority of HERVs are transcriptionally inactive, due to the accumulation of multiple mutations during evolution [11]. About 90% of HERV exist as solo LTRs because of loss of internal gene sequences by homologous recombination [10]. In addition, their activation is inhibited, for example by TRIM-5α (tripartite motif 5α) or APOBEC3 (apolipoprotein B mRNA-editing catalytic polypeptide) [11], [12]. Silencing of the expression of HERV is also due to hypermethylation of their promoter regions [13], [14]. However some HERVs (mainly belonging to the HERV-K group) are still active and capable of expressing, both at the mRNA and protein levels [15]. It is particularly the case of HERV-K113, which is able to produce viral particles, although without the possibility of infecting new cells [16].

Human endogenous retroviruses were suspected to be related to autoimmune diseases, including multiple sclerosis [17]–[21] and rheumatoid arthritis [22]–[24], psoriasis [25], breast cancer [26], viral infections [27]–[29] and even schizophrenia [14], [17], [30]. It seems to be very important to look into a possible link between endogenous retroviruses present in the human genome as a result of ancient infections and exogenous viruses that attack us nowadays. The latter include human T-cell lymphotropic viruses type 1 and 2 (HTLV-1, HTLV-2) and of course HIV. Some researchers have pointed to the possibility of transcomplementation of HIV and HERV proteins during HIV infection (using HERV envelope, regulatory proteins or enzymes such as dUTPase or protease) [31]–[35]. Some reports indicated increasing HERV transcription [15], [36] and humoral and cellular response to HERV proteins during HIV-1 infection [12], [15], [31], [37]. These observations were often related to retroviral elements belonging to the HERV-K family.

The insertionally polymorphic sequences HERV-K113 and HERV-K115 were found in the genome in one copy located on chromosome 19p13.11 and 8p23.1, respectively. They share more than 95% nucleotide sequences similarity and the same genome organisation [2], [26], [38]. It is estimated that HERV K113 was incorporated into the genome about 800 thousand years ago and HERV-K115 is present in human genome even longer – about 1 million years [39]. Their frequencies were different depending on the geographical region, with the highest prevalence in Africans (mean 22% for HERV-K113 and 27% for HERV-K115), about 2–3% in Europeans and almost not reported in Papua New Guineans [39].

The aim of this study was firstly to determine the prevalence of sequences HERV-K113 and HERV-K115 in a population of the Lower Silesia region of Poland (unexposed to HIV-1), and secondly to compare HIV-1-infected people and exposed but uninfected (EU) individuals. To our knowledge, this is the first study of its kind to be carried out. In particular, we compared prevalence of endogenous retroviral sequences in groups of people exposed to HIV but seronegative (EU) and HIV-1 positive to find any relationship between them and HIV-1 infection.

## Materials and Methods

### Studied Groups

HERV-K113 and HERV-K115 presence was determined in the Lower Silesia region of Poland. Three groups of individuals were analyzed. The first, control group, consisted of 303 healthy individuals, not exposed to HIV-1. In the second one there were 121 people repeatedly exposed to HIV-1 but uninfected (EU) with different routes of exposure: long-lasting sexual partnership with HIV-1 infected people (33 heterosexual and 2 homosexual), 84 intravenous drug users with at least 1.5 years addiction history (1.5 to 36 years, average 13.7 years) and 2 children of seropositive mothers, uninfected despite lack of vertical infection prevention. The low sample size of homosexual EU group is due to the difficulties in identification of these patients. The third group (HIV(+)) consisted of 470 HIV-1 positive patients of the Department and Clinic of Infectious Diseases, Hepatology and Acquired Immune Deficiencies, Wroclaw Medical University. HIV infection was connected with the homosexual (35 individuals) or heterosexual route (61), intravenous drug use (359), vertical transmission during delivery (3), blood transfusion (1), blood products application (1), and an unknown way (10) of HIV-1 infection. All participants provided their written informed consent to participate in this study, according to the Helsinki Declaration, and the study was approved by the Commission of Bioethics at Wroclaw Medical University (number of permission: KB-182/2005).

The detailed characteristics of the studied groups are given in Table 1.

**Table 1:** Characteristics of studied groups.

Groups	Control [HIV(−)]	EU [HIV(−)]	HIV(+)
Number of individuals [n]	303	121	470
Age range (mean) [years]	5–71 (33.6)	3–50 (30.5)	0–59 (30.7)
Sex (female/male) [n (%)]	159/144 (52.5/47.5%)	41/80 (33.8/66.2%)	158/312 (33.6/66.4%)
Homosexual exposure [n (%)]	NA	2 (1.7%)	35 (7.5%)
Heterosexual exposure [n (%)]	NA	33 (27.2%)	61 (13.0%)
Intravenous drug users [n (%)]	NA	84 (69.4%)	359 (76.4%)
Mother to child transmission [n (%)]	NA	2 (1.7%)	3 (0.6%)
Blood transfusion [n(%)]	NA	0 (%)	1 (0.2%)
Blood products [n(%)]	NA	0 (%)	1 (0.2%)
Unknown route of infection [n(%)]	NA	0 (%)	10 (2.1%)
HCV(+) [n (%)]	NT	77 (63.6%)	356 (75.7%)
HCV(−) [n (%)]	NT	39 (32.2%)	109 (23.2%)
No data for HCV co-infection [n (%)]	NT	5 (4.2%)	5 (1.1%)

NA – not applicable; NT – not tested.

EU – exposed uninfected.

### HERV Detection

Detection of sequences of endogenous retroviruses was performed with the PCR method. Genomic DNA of examined individuals was isolated from peripheral EDTA-anticoagulated blood. DNA was extracted using protease K digestion and purification on QIAamp silica-gel columns (QIAamp® DNA Blood Mini Kit; Qiagen, Germany). Then, PCRs for detection of HERV-K113 (according to Moyes et al., 2005) [19] and HERV-K115 (based on Burmeister et al., 2004) were performed [26]. PCRs were done in 10 µl reaction volume in a T3000 thermocycler (Whatman Biometra, Germany) using Taq polymerase (Polgen, Poland). PCR primers used in mentioned reactions (Symbios, Straszyn, Poland) are presented in Table 2.

**Table 2:** Primer sequences for HERV-K113 and HERV-K115 PCR.

Detection	Primer	Sequences
HERV-K113	K113-F1	5′ - GCA TGG GGA GAT TCA GAA CC - 3′
	K113-R1	5′ - CAT GTT TCC TGT CTG CCC AC - 3′
	K113-LTR-F1	5′ - GGA CCT GCG GGC AGC AAT ACT G - 3′
	K113-LTR-R1	5′ - TCG GGA TCT CTC GTC GAC TTG TCC - 3′
HERV-K115	K115-F2	5′ - CCG CAC CTA GTC AAC TTA GC - 3′
	K115-R2	5′ - TCA GTT CCC GAT TTT CTG CC - 3′
	K115-LTR-F2	5′ - GGA TCC TCC ACA TGC TGA A - 3′
	K115-LTR-R2	5′ - TCT CAA GGC AGA AGA ATT TTT CTT AG - 3′
	K115-PROV-R2	5′ - CTT CAC CCT AGA GAA AAG CCT CC - 3′

1 Sequences from Moyes et al., 2005 [19].

2 Sequences from Burmeister et al., 2004 [26].

**Figure 1:**
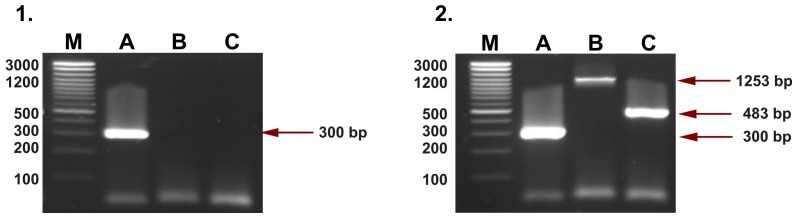
Representative gels showing results of PCR HERV-K113 detection in this work. 1– homozygous negative individual (lack of HERV-K113 insertion); 2– heterozygous individual (retroviral insertion in one chromosome). An individual with HERV-K113 on both chromosomes was not detected. M – size standard; A – lines corresponding to the preintegration site (reaction A, 300 bp product); B – lines for HERV-K113 5′LTR and provirus fragment (reaction B, 1253 bp product); C – lines for HERV-K113 3′LTR (reaction C, 483 bp product).

Three separate reactions were required for detection of HERV-K113 for each sample. The first reaction (A), using the pair of primers K113-F and K113-R (Table 2), produced a 300 bp fragment, corresponding to the insertion site of the endogenous retrovirus (no insertion). The second one (B), using the pair of primers K113-F and K113-LTR-R (Table 2), was used to detect the 5′LTR sequence and HERV-K113 provirus fragment (1253 bp fragment). The amplification with primers pair LTR-K113-F and K113-R (Table 2) (reaction C) was used to obtain a 483 bp fragment corresponding to the 3′LTR of HERV-K113. A positive result of the last two reactions showed the presence of HERV-K113 in the genome of studied individuals. The PCR conditions for HERV-K113 detection were as follows: 3 min at 94°C; 40 cycles: 1 min at 94°C, 1 min at 55°C (for reactions A and C) or 1 min at 59°C (for reaction B); 1 min at 72°C and the final extension step of 5 min at 72°C. PCR products were separated in 2% agarose (AppliChem, Darmstadt, Germany) gel stained with ethidium bromide (0.5 µg/ml) and visualised in UV light (Fig. 1).

**Figure 2:**
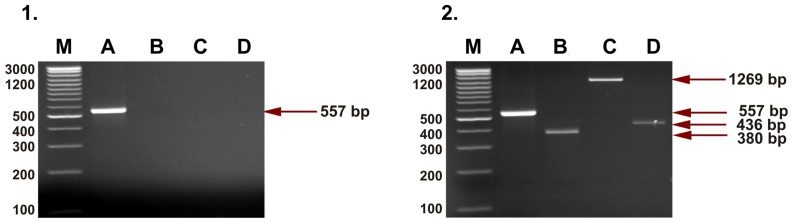
Agarose gels of PCR products of HERV-K115 detection. 1– homozygous negative individual (lack of HERV-K115 insertion); 2– heterozygous individual (retroviral insertion in one chromosome). No homozygous individual with retroviral HERV-K115 insertion on both chromosomes was detected. A – lines for preintegration site (reaction A, 557 bp product); B – lines for HERV-K115 5′LTR (reaction B, 380 bp product); C – lines corresponding to the provirus HERV-K115 fragment (reaction C, 1269 bp product); D – lines for HERV-K115 3′LTR (reaction D, 436 bp product).

Four separate reactions were performed for detection of HERV-K115 for each sample. The first one (A), with the pair of primers K115-F and K115-R (Table 2), was performed to detect the “wild-type” sequence (without HERV-K115 in the genome, 557 bp). The second one (B), with K115-F and K115-LTR-R primers (Table 2), let us find a fragment of 5′LTR (380 bp). The third one (C) with K115-F and K115-PROV-R primer set (Table 2) served in detection of provirus presence in the tested genome (1269 bp band). The fourth reaction (D) required amplification of K115-LTR-F and K115-R primers and gave PCR fragment for 3′LTR (436 bp). A positive result of the last three reactions indicated the presence of HERV-K115 in the genome of tested people. PCRs were performed as follows: 3 min at 94°C; 30 cycles: 30 s at 94°C, 30 s at 54°C, 30 s at 72°C and a final extension set of 10 min at 72°C. Amplification products were analysed in 2% ethidium bromide stained agarose gel and visualised in UV light (Fig. 2).

### Statistical Analysis

The distribution of HERV-K113 and HERV-K115 was calculated in control (n = 303), EU (n = 121) and HIV(+) (n = 470) groups. Differences between groups were analysed using two-sided Fisher exact test. A *p* value of less than 0.05 was taken to be significant. A conceivable relation between studied endogenous retroviruses and HIV infection was evaluated using logistic regression in the general linear model’s scheme. The comparison of genetic and non-genetic factors in EU (n = 114) and HIV(+) (n = 452) groups was performed. Beside the impact of HERV-K113 and HERV-K115, the role of route of exposure to HIV (homosexual, heterosexual and intravenous drug use), sex and age of patients, and HCV carrying were also tested. Variables were included in the model with p<0.05. All statistical analyses were performed using the platform R-CRAN version 2.8.1 (www.r-project.org).

## Results

Prevalence of HERV-K113 and HERV-K115 in the Lower Silesia population of Poland was 11.8% and 7.92%, respectively (control group, n = 303; Table 3). No homozygous individual or solo LTR was detected in any of the tested groups. Genotype distributions of both HERVs in control as well as in EU and HIV(+) groups were compatible with the Hardy-Weinberg principle. Prevalence of HERV-K113 and HERV-K115 in the EU group (exposed uninfected individuals; n = 121) was 8.26% and 5.71% respectively. In the HIV(+) group (n = 470) these sequences were detected in 12.98% for HERV-K113 and 7.23% for HERV-K115. There were no statistically significant differences between mentioned groups and the control group (p>0.05).

**Table 3:** Distribution of HERV-K113 and HERV-K115 in control group, exposed to HIV but uninfected (EU) and HIV-positive patients.

Group	HERV-K113				HERV-K115			
	Lack of HERV-K113 [n]	HERV-K113 (+) [n]	HERV-K113(+) [%]	P-value	Lack of HERV-K115 [n]	HERV-K115 (+) [n]	HERV- K115(+) [%]	P-value
Control (n = 303)	267	36	11.88	0.546	279	24	7.92	0.773
EU (n = 121)	111	10	8.26	0.475	114	7	5.79	0.681
HIV(+) (n = 470)	409	61	12.98	0.208	436	34	7.23	0.658

To analyse the potential connection between studied endogenous retroviral elements and HIV infection we compared HERV-K113 and K115 distributions in the group of HIV-infected patients (n = 452) and in the group of patients exposed repeatedly for a long time to HIV, but seronegative (EU, n = 114). We also took into account other factors: age, sex, type of exposure to HIV and HCV carrying. All mentioned factors were analysed by logistic regression. The results of endogenous retroviruses detection in EU and HIV(+) groups with reference to sex, mode of exposure and HCV coinfection are presented in Table 4. Among tested agents only HCV co-infection (OR = 12.90; CI95% 4.69–35.48; p = 0.00002) and homosexual exposure (OR = 7.69; CI95% 1.88–31.48; p = 0.007017) were found to be factors increasing susceptibility to HIV infection, as we reported previously [40]. The homosexual EU group was very limited (n = 2) so it requires inclusion of more patients to confirm that conclusion. We found no relation between studied HERVs and HIV infection (p>0.05).

**Table 4:** Characteristics of HIV-infected and exposed uninfected patients included in analysis of HERV-K113 and HERV-K115 connection with HIV infection.

Characteristics of studied groups		EU					HIV(+)				
		n (%)	HERV-K113[n (%)]	No HERV-K113[n (%)]	HERV-K115[n (%)]	No HERV-K115[n (%)]	n (%)	HERV-K113[n (%)]	No HERV-K113[n (%)]	HERV-K115[n (%)]	No HERV-K115[n (%)]
Number of individuals		114 (100.0)	10 (8.8)	104 (91.2)	7 (6.1)	104 (93.9)	452 (100.0)	57 (12.6)	395 (87.4)	33 (7.3)	419 (92.7)
Sex [n (%)]	female	36 (31.6)	4 (11.1)	32 (88.9)	1 (2.8)	35 (97.2)	151 (33.4)	25 (16.6)	126 (83.4)	12 (7.9)	139 (92.1)
	male	78 (68.4)	6 (7.7)	72 (92.3)	6 (7.7)	72 (92.3)	301 (66.6)	32 (10.6)	169 (89.4)	21 (7.0)	280 (93.0)
Mode of infection[n (%)]	homosexual	2 (1.7)	0	2 (100.0)	0	2 (100.0)	35 (7.7)	4 (11.4)	31 (88.6)	5 (14.3)	30 (85.7)
	heterosexual	28 (24.6)	2 (7.1)	26 (96.9)	1 (3.6)	27 (96.4)	61 (13.5)	11 (18.0)	50 (82.0)	4 (6.6)	57 (93.4)
	Intravenous drugusers (IDU)	84 (73.7)	8 (9.5)	76 (90.5)	6 (7.0)	78 (93.0)	356 (78.8)	42 (11.8)	314 (88.2)	24 (6.7)	332 (93.3)
HCV infection [n (%)]	−	37 (32.5)	3 (8.1)	34 (91.9)	2 (5.4)	35 (94.6)	98 (21.7)	15 (15.3)	83 (84.7)	5 (5.1)	93 (94.9)
	+	77 (67.5)	7 (9.1)	70 (90.9)	5 (6.5)	72 (93.5)	354 (78.3)	42 (11.9)	312 (88.1)	28 (7.9)	326 (92.1)

## Discussion

Literature shows an increasing amount of information about the possible role of endogenous retroviruses in health and disease. Some of those retroelements are involved in human placenta development (HERV-W, HERV-FRD) [9], [41], in development and differentiation of normal tissue during embryogenesis [42], and in prevention of fetus rejection by the mother immune system [43]. Another beneficial aspect of HERVs presence in the human genome could be interference with exogenous retroviruses by blocking their receptors with protein Env or interference with virus replication by antisense RNA, which was shown in the case of murine endogenous retroviruses [4]. HERVs were found to be related to various diseases, such as autoimmune diseases [14], [19]–[23], [44], cancer [26], [45], [46], and even mental disorders [17], [24], [30]. It was not clear whether HERVs were causative agents of the disease, or whether their expression during disease development occurred only accidentally.

As HERVs are not only a genetic ballast, it is important to identify their occurrence in the population, especially with regard to sequences capable of expression as HERV-Ks. In the present work we focused on determining the occurrence of HERV-K113 and HERV-K115 sequences in the Polish population. We did not test sequence variation of these retroviruses in a population. Genotyping in the control group consisting of 303 healthy people revealed that provirus HERV-K113 was present in 36 individuals (11.88%) and HERV-K115 in 24 people (7.92%) (Table 3). There were no homozygous individuals in the studied group in the case of both analysed sequences. Prevalence of these sequences is ethnically dependent. HERV-K113 prevalence was between 0 (Papua New Guinea) and 36% (Kenya) [19], [39]. In Europe the frequency of HERV-K113 insertion was between 0 and 4.2% [19], [26], [39]. Krzyształowska-Wawrzyniak et al. determined HERV-K113 frequency for the Polish population as 8.05% [44]. Against this background HERV-K113 frequency (11.88%) determined in our study was much higher and rather close to that given by Burmeister et al. for the German population (12.8%) [26]. HERV-K115 reported prevalence was found to be between 0 (Papua New Guinea) and 43.3% (Côte d’Ivoire) [19], [39]. In Europe Jha et al. reported mean HERV-K115 insertion frequency as 3% [39]. We determined HERV-K113 and HERV-K115 frequency in the Polish population for the first time. As for HERV-K113, prevalence of HERV-K115 in the Lower Silesia region of Poland was higher than the average European frequency and similar to that noted for the German population (7.92% vs 9.8%) [26].

In our study we wanted to find out if there are any differences in HERV-K113 and HERV-K115 presence between infected and uninfected individuals exposed to HIV. According to our results, frequencies of the HERVs insertion were lower in the EU group (8.26% for HERV-K113 and 5.79% for HERV-K115) than in HIV-positive patients (12.98% for HERV-K113 and 7.23% for HERV-K115). But logistic regression revealed that these differences were not statistically significant.

There are many suggestions about the potential relationship between HERV presence in the genome and the replication of HIV. The virus was found to be able to use HERV-W envelope glycoprotein [4], [32]. In addition, it was suggested that HIV could upregulate HERV-K structural genes, as was found by Contreras-Galindo et al. [15], [31], [47], [48]. They showed the presence of mRNA of HERV-K (highly homologous to HERV-K108, K109, K113, and K115) in plasma in more than 95% of people infected with HIV, and only in 7% of uninfected people. The expression level of HERV-K in HIV-positive patients was correlated with the level of HIV viraemia [15]. High levels of HERV-K gag and env mRNA in HIV-positive individuals were also reported by Ormsby et al. [49]. The confirmation of the impact of HIV on HERVs expression was the finding of humoral and cellular responses against the structural proteins of HERV-K in people with HIV [12], [15], [37]. Drugs used in the antiretroviral therapy were shown to restrict but not inhibit HERV expression. It indicated the possibility of the transactivation of HERV-K proviruses by the HIV proteins, which were the products of the latent virus in monocytes and CD4+ T cells [50]. HIV can also induce expression of HERV-K protease PR in the HIV-specific CD4+ T-lymphocytes [33]. Studies *in vitro* revealed that the protease, a product of HERV-K10, had the ability to cut the capsid and matrix proteins of HIV; a cut was made in the correct places recognized by HIV protease. Although HERV protease exhibited activity approximately 20-fold lower than HIV PR, it was found to be particularly resistant to HIV protease inhibitors [34]. However, studies have shown that Gag and Pol precursor cutting by PR HERV-K10 did not give correct products, and the introduction of the PR HERV-K10 gene to a HIV clone without its own PR did not result in restoring the infectivity [33]. On the other hand, it was also found that the HERV-K10 protease was able to replace the functional wild strains of HIV PR. However, no data that would preclude this possibility in the case of HIV protease inhibitors have been obtained, or in the case of strains which have developed as a result of multi-drug resistance mutations [33]. It was shown that endogenous retroviruses belonging to the family HERV-K (HERV-K10) encoded the functional homolog of HIV regulatory genes *rev* and HTLV *rex* (named *K-rev*, *Corfe* or *Rec*) [35], [37], [51]. The protein Rec (weight 14 kDa), as well as HIV Rev, was localized in the nucleus, and its function was to transport the uncut HERV-K mRNA from the nucleus to the cytoplasm. Interaction with CRM-1 (nuclear transport factor) and RRE (Rev response element) within the HERV-K mRNA (K-RRE) occurred during transport (similar to the transport of HIV mRNA) [35], [37]. It suggested interaction between the random K-RRE and Rev of HIV [37]. Genes encoding Rec are present in the polymorphic sequences of HERV-K113 and HERV-K115 [15], [19], [36], [52]. Up until now, no interactions between Rec proteins and the RRE region of HIV have been found [37], but it cannot be ruled out. According to Monde et al., HERV-K Gag can reduce infectivity of HIV-1 and the release of this virus from cells [53]. Thus, it appears that the presence of endogenous retroviruses in the genome may in some way affect the course of HIV infection but probably not susceptibility to infection.

## Conclusions

Prevalence of endogenous retroviral sequences HERV-K113 and HERV-K115 in the Polish population (11.88% and 7.92%, respectively) was higher than calculated for the majority of European populations. Since there were no statistically significant differences found between HIV(+), EU and control groups, no relation between studied HERVs and HIV infection was shown.
